# Parasympathetic Nervous System Dysfunction, as Identified by Pupil Light Reflex, and Its Possible Connection to Hearing Impairment

**DOI:** 10.1371/journal.pone.0153566

**Published:** 2016-04-18

**Authors:** Yang Wang, Adriana A. Zekveld, Graham Naylor, Barbara Ohlenforst, Elise P. Jansma, Artur Lorens, Thomas Lunner, Sophia E. Kramer

**Affiliations:** 1 Section Ear & Hearing, Dept. of Otolaryngology-Head and Neck Surgery and EMGO Institute for Health and Care Research, VU University medical center, Amsterdam, The Netherlands; 2 Eriksholm Research Centre, Oticon A/S, Snekkersten, Denmark; 3 Department of Behavioural Sciences and Learning, Linköping University, Linköping, Sweden; 4 Linnaeus Centre HEAD, The Swedish Institute for Disability Research, Linköping and Örebro Universities, Linköping, Sweden; 5 MRC/CSO Institute of Hearing Research, Scottish Section, Glasgow, United Kingdom; 6 Medical Library, VU University Amsterdam, Amsterdam, the Netherlands; 7 Institute of Physiology and Pathology of Hearing, Warsaw, Poland; 8 Department of Clinical and Experimental Medicine, Linköping University, Linköping, Sweden; UMR8194, FRANCE

## Abstract

**Context:**

Although the pupil light reflex has been widely used as a clinical diagnostic tool for autonomic nervous system dysfunction, there is no systematic review available to summarize the evidence that the pupil light reflex is a sensitive method to detect parasympathetic dysfunction. Meanwhile, the relationship between parasympathetic functioning and hearing impairment is relatively unknown.

**Objectives:**

To 1) review the evidence for the pupil light reflex being a sensitive method to evaluate parasympathetic dysfunction, 2) review the evidence relating hearing impairment and parasympathetic activity and 3) seek evidence of possible connections between hearing impairment and the pupil light reflex.

**Methods:**

Literature searches were performed in five electronic databases. All selected articles were categorized into three sections: pupil light reflex and parasympathetic dysfunction, hearing impairment and parasympathetic activity, pupil light reflex and hearing impairment.

**Results:**

Thirty-eight articles were included in this review. Among them, 36 articles addressed the pupil light reflex and parasympathetic dysfunction. We summarized the information in these data according to different types of parasympathetic-related diseases. Most of the studies showed a difference on at least one pupil light reflex parameter between patients and healthy controls. Two articles discussed the relationship between hearing impairment and parasympathetic activity. Both studies reported a reduced parasympathetic activity in the hearing impaired groups. The searches identified no results for pupil light reflex and hearing impairment.

**Discussion and Conclusions:**

As the first systematic review of the evidence, our findings suggest that the pupil light reflex is a sensitive tool to assess the presence of parasympathetic dysfunction. Maximum constriction velocity and relative constriction amplitude appear to be the most sensitive parameters. There are only two studies investigating the relationship between parasympathetic activity and hearing impairment, hence further research is needed. The pupil light reflex could be a candidate measurement tool to achieve this goal.

## Introduction

**Fig 1:**
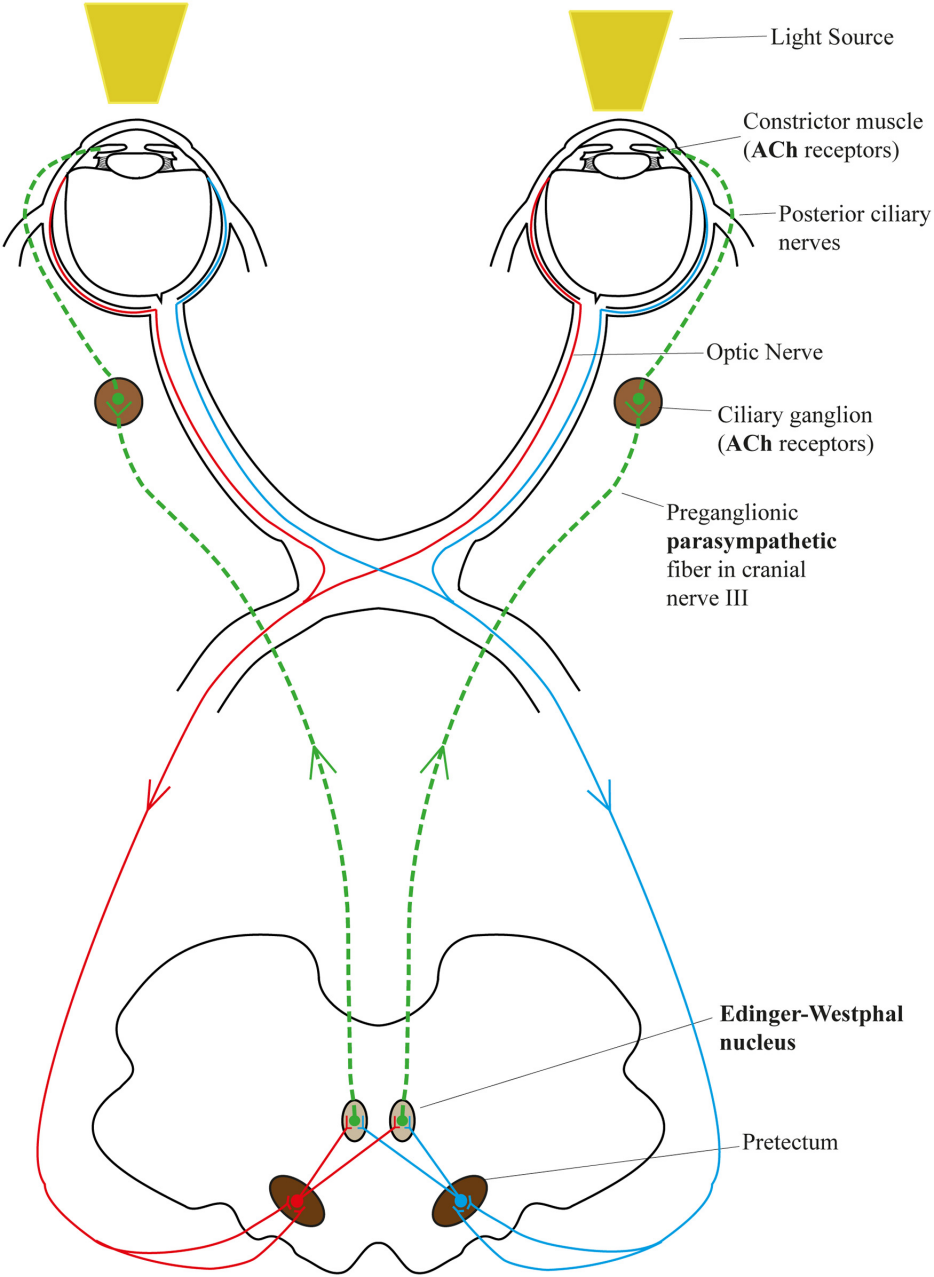
Pupil light reflex pathway. Red and blue lines represent the afferent pathway, the ganglion cell axons project to the pretectal region of the midbrain; green line represents the efferent pathway, the signal transmits from preganglionic parasympathetic fiber to ciliary ganglion, finally to the constrictor muscles through the short posterior ciliary nerve.

The pupil light reflex (PLR) is the reflex whereby a change in pupil size occurs in response to an increase of light intensity falling on the retina [1]. Under the direct control of the autonomic nervous system (ANS), the pupil light reflex reflects the balance between the Sympathetic Nervous System (SNS) and the Parasympathetic Nervous System (PNS), which are the two main branches of the autonomic nervous system. The constrictor muscle (sphincter muscle) of the pupil decreases the pupil diameter under the control of the ciliary ganglion. The pathway of that process is illustrated in Fig 1 and functions as follows: light falling on the retina(s) leads to increased neural activity in the pretectal regions and stimulation of the Edinger-Westphal nucleus, where preganglionic parasympathetic neurons are activated and innervate the ciliary ganglion. These in turn command the constrictor muscle(s) to tighten and this leads to pupil constriction [1] (see Fig 1). Both the ciliary ganglion and constrictor muscles contain Acetylcholine (ACh) receptors, which is the main neurotransmitter of the PNS.

**Fig 2:**
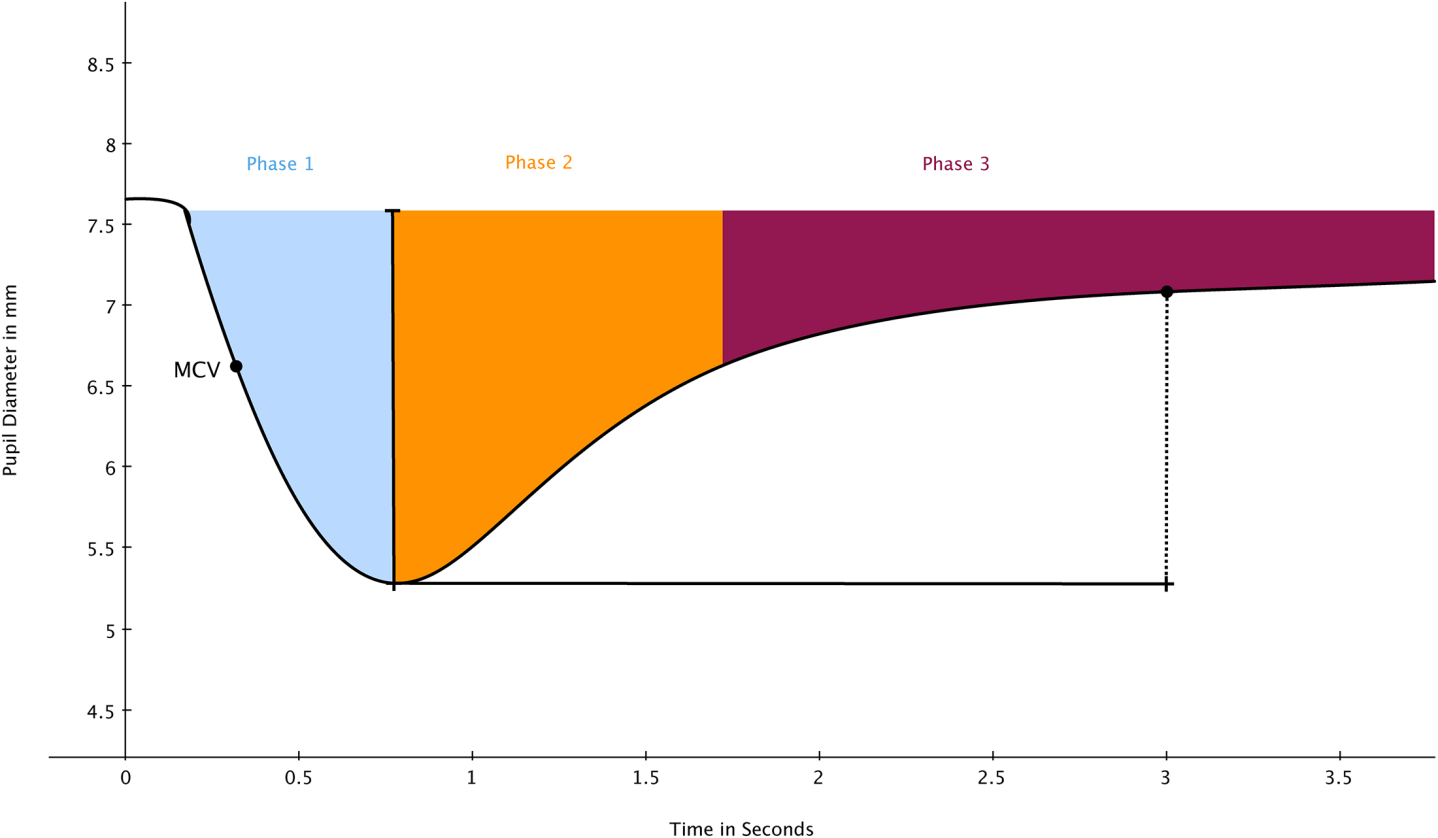
demonstration of one pupil light reflex on pupillometry. The light onsets at the ‘0’ point; Phase 1 is a fast constriction mainly controlled by PNS; Phase 2 is a fast redilation under the control of both PNS and SNS; Phase 3 is a slow redilation phase, predominantly controlled by SNS activity.

Fig 2 is a schematic illustration of a pupil light reflex. The light stimulus onsets at time zero, and the constriction starts with a latency of about 200 ms. A standard light reflex contains three phases, 1) a fast constriction, followed by 2) a fast redilation of the pupil, and then 3) a slow redilation where the pupil recovers to its original size. The pupil constricts rapidly in the beginning, then after it reaches its maximum constriction velocity (point MCV in Fig 2), the constriction becomes slower until the minimum diameter is reached. The latency of constriction, maximum constriction velocity, and absolute constriction amplitude (difference between baseline and minimum pupil diameter) are the usual parameters used in pupil light reflex analysis and will be more closely defined later in the result section. In general, reduced PNS activity is characterized by longer constriction latency, slower maximum constriction velocity and smaller constriction amplitude of the pupil light reflex [2]. According to Loewenfeld and Lowenstein [2], PNS plays a dominant role during the pupil constriction phase, while SNS contribution is negligible. On the other hand, both PNS and SNS innervate the pupil in the beginning of the redilation phase. Thus, in theory, observing the constriction part of the pupil light reflex provides an indicator of PNS activity uncontaminated by SNS activity.

Studies using the pupil light reflex to evaluate PNS activity are well established. For example, it has been used to test patients with clinical signs of PNS dysfunction, especially related to cholinergic deficiencies like Parkinson’s Disease [3], Alzheimer’s Disease [4] and Diabetes Mellitus [5]. Moreover, researchers have also tested pupil light reflex on psychiatric disorders like Schizophrenia [6] and anxiety disorder [7]. In addition, the pupil light reflex has been found to be sensitive to aging [8], smoking [9] and gender [10].

Many studies about the pupil light reflex are available nowadays, but to evaluate its sensitivity in the evaluation of PNS activity, especially for PNS dysfunction caused by PNS related conditions, a thorough review of more recent pupil light reflex studies is needed. Certainly Loewenfeld’s book [2] includes numerous information about the history and literature in this field, but it was published more than 20 years ago, and some of the information may be outdated.

More fundamentally, the autonomic nervous system plays a direct role in the human stress response. SNS is in control of the so-called ‘fight or flight’ response to stressors. Sympathetic activation, releasing noradrenaline from adrenal glands, is the typical reaction when the body faces a stressful situation [11]. In contrast to SNS’s ‘excitatory’ role, PNS is in charge of the ‘rest and digest’ response. PNS activation causes a range of reactions that help the body to restore energy and recover from stress, including maintenance of homeostasis, slowing of heart rate, and constriction of the gut and salivary glands through the release of ACh [12]. Autonomic response to physical or emotional stressors usually encompasses increased activity in the SNS branch accompanied by decreased activity in the PNS branch [13].

Hearing impairment may have adverse effects on daily life functioning. Multiple studies suggest that hearing impairment is associated with psychosocial problems like depression or loneliness [14, 15, 16]. Associations between hearing impairment and increased levels of stress are also frequently reported [16, 17]. Acute stress may be evoked by physical (e.g. pain, noise exposure) or psychological stressors (e.g. expectations or experience of failure, negative reactions from peers towards the hearing impairment) [18]. Long-term stress may emerge when people are frequently exposed to stressful situations [19]. Stress in hearing impaired adults might result in withdrawal from major social roles, especially occupational roles [16, 20, 21]. At the same time, some evidence also suggests that emotional and physical stress could lead to hearing problems [18], i.e. with a reversed direction of causation.

Sympathetic innervation to the inner ear is well documented. SNS innervates the cochlea by regulating cochlear blood flow via the local adrenergic α-receptors. In addition, inner ear function is under the influence of sympathetic activity via the central noradrenergic pathway [18]. In contrast, the contribution of the PNS branch to the cochlear innervation is relatively unexplored [22], although there is some evidence suggested by Ross and Jones [23] about the possible parasympathetic innervation of the inner ear. Thus, evaluation of stress level from the perspective of autonomic function may play an important role in a more comprehensive understanding of the mechanisms and consequences of hearing impairment. Up until now, most studies on the relationship between the ANS and hearing impairment have focused on monitoring SNS activities. For instance, differences between people with normal hearing and impaired hearing were found through measurements of pupil dilation [24, 25], cortisol level [26] and skin conductance [27]. By contrast, there are very few studies making direct connections between PNS activity and hearing impairment, although there is some evidence suggesting that the level of stress related to hearing impairment is negatively correlated to the activity of the PNS [17, 27]. The second aim of the present study was to gain a better understanding of the relationship between PNS and hearing impairment by collating the available evidence in the research area. Performing a systematic review to find all potential connections between PNS and hearing impairment is necessary for such understanding. It may be noted that some forms of autonomic system neuropathy and auditory-nerve neuropathy might co-exist in metabolic diseases such as diabetes mellitus, without any causal link. Thus a complete description of the mechanisms potentially connecting PNS and hearing impairment should include the elimination of such alternative explanations for apparent correlations.

This paper presents a systematic review of the existing literature, performed in conformance with the PRISMA statement [28]. We first reviewed the extent to which the pupil light reflex is a sensitive method to evaluate PNS dysfunction caused by PNS-related diseases. The second objective was to gain insight into the possible relationships between hearing impairment and PNS, and to seek any evidence that people with hearing impairment might show abnormal PNS activity. If the first two objectives were fullfilled with affirmative results, the next logical step would be to examine relations between pupil light reflex and hearing impairment directly. Therefore our search strategies also included this pairing. The ultimate purpose of this study was thus to pave the way for future studies testing the pupil light reflex on hearing impaired subjects.

## Methods

The systematic review was performed in accordance with the guidelines provided by the PRISMA statement [28]. The supporting PRISMA checklist is available as supporting information (see S1 Appendix).

### Eligibility criteria

**Fig 3:**
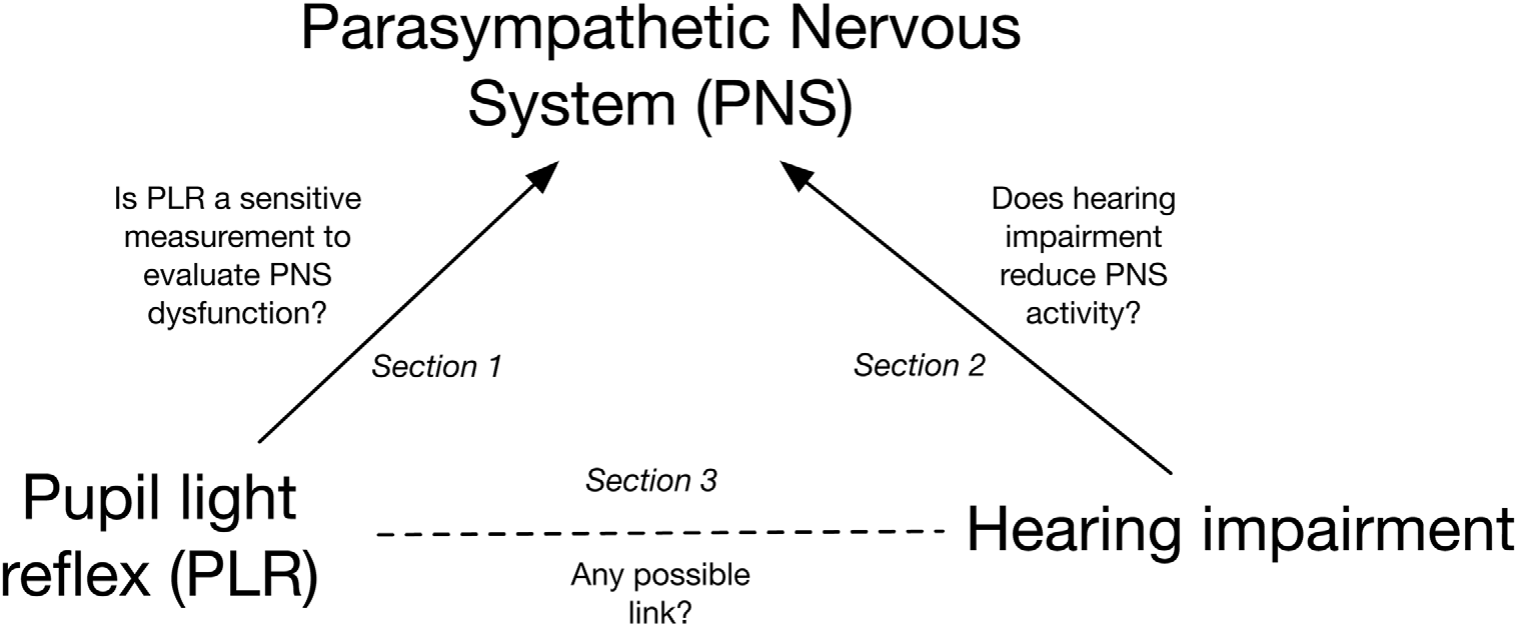
Structure of the review.

This study was divided into three sections, describing the evidence for the relationships between: PLR and PNS dysfunction, hearing impairment and PNS, and hearing impairment and PLR (see Fig 3). In the literature search that identified the potential studies, eligibility criteria were separately implemented for each section. The PICOS (Participants, Interventions, Comparators, Outcomes, and Study design) [28] approach was used to aid development of the eligibility criteria for each section.

**Section 1: Pupil Light Reflex and Parasympathetic Nervous System Dysfunction**

***P*** *(types of participants)*: Participants of any age with recognized parasympathetic-related conditions (Parkinson, diabetes, Alzheimer, autonomic neuropathy, aging etc.) were considered. As no definitive list of parasympathetic-related conditions was forthcoming, the list of conditions for inclusion was assembled retrospectively after the overall search was completed, as follows: for each condition which featured in the results of search #6 (see Table 1 below), a Google Scholar search was run with ‘Parasympathetic’ and the name of the given condition. If inspection of the results indicated widespread recognition of a relation between PNS dysfunction and the condition in question, that condition was included in the list. Studies of participants with diseases of the visual system were excluded due to the possibility of confounding factors in PLR results. The keywords ‘parasympathetic’ or ‘cholinergic’ must be mentioned in the title or abstract or keywords.

**Table 1:** Search steps and results of PubMed.

Search	PubMed Query 30-10-2014	Items found
#8	Search **#7 NOT ("animals"[MeSH Terms] NOT "humans"[MeSH Terms])**	1155
#7	Search **#4 OR #5 OR #6**	1701
#6	Search **#2 AND #3**	328
#5	Search **#1 AND #3**	1346
#4	Search **#1 AND #2**	27
#3	Search **"Parasympathetic Nervous System"[Mesh] OR "Acetylcholine"[Mesh] OR "parasympathetic"[tiab] OR "cholinergic"[tiab] OR "Vagus Nerve"[tiab] OR "Nerves vagus"[tiab] OR "ciliary ganglion"[tiab] OR "ganglion ciliare"[tiab] OR "ganglion opticum"[tiab] OR "optic ganglion"[tiab]** Filters: **Publication date to 2014/10/24**	132367
#2	Search **"Reflex, Pupillary"[Mesh] OR "Pupillary reflex"[tiab] OR "pupil reaction"[tiab] OR "pupil reflex"[tiab] OR "light reflex"[tiab] OR "pupillary reaction"[tiab] OR "pupillary reactivity"[tiab] OR "pupil reactivity"[tiab] OR "pupillary response"[tiab] OR "pupil response"[tiab] OR "dynamic pupil"[tiab] OR "flash response"[tiab]** Filters: **Publication date to 2014/10/24**	3570
#1	Search "Hearing"[Mesh:NoExp] OR "Hearing disorders"[Mesh] OR "Persons With Hearing Impairments"[Mesh] OR "hearing loss"[Mesh] OR Presbyacusis[tiab] OR Presbyacusia[tiab] OR Presbycusis[tiab] OR hypoacusis[tiab] OR Deaf*[tiab] OR (Hearing[tiab] AND (condition*[tiab] OR disabilities[tiab] OR disability*[tiab] OR disabled*[tiab] OR disorder*[tiab] OR handicap*[tiab] OR impair*[tiab] OR loss[tiab] OR Problem*[tiab])) Filters: Publication date to 2014/10/24	108076

***I*** *(types of intervention)*: Pupil (pupillary) light reflex/ Pupil (pupillary) light reaction/ Pupil (pupillary) light response/Pupil (pupillary) response only caused by light stimuli. Studies measured with ‘pupil cycle time’ were excluded, because this measurement is different from standard PLR. Drug studies using PLR as a testing tool for autonomic function were excluded, as these studies assume that PLR reflects PNS function and therefore do not test this hypothesis.

***C*** *(types of control group)*: Adults with normal parasympathetic functions or proven differences in parasympathetic functioning.

***O*** *(types of outcome measures)*: PLR parameters and their relationships (correlations) to the PNS activity.

**Section 2: Hearing Impairment and Parasympathetic Nervous System**

***P*** *(types of participants)*: People with hearing impairment. Hearing impairment was defined as loss of hearing ability. Studies on Deaf people without any residual hearing who use sign language and lip-reading only were excluded. People whose hearing ability was restored by hearing aid(s) or cochlear implant(s) were also considered as hearing impaired. No restriction was applied to the severity of hearing impairment.

***I*** *(types of intervention)*: Physiological measurements of PNS activity like heart rate variability, skin conductance and blood pressure.

***C*** *(types of control group)*: Adults with normal hearing.

***O*** *(types of outcome measures)*: PNS activity, measured by physiological methods other than the PLR and their relationships (correlations) with hearing status.

**Section 3: Hearing Impairment and Pupil Light Reflex**

***P*** *(types of participants)*: see *P(types of participants)* in Section 2.

***I*** *(types of intervention)*: Pupil (pupillary) light reflex/ Pupil (pupillary) light reaction / Pupil (pupillary) light response / Pupil (pupillary) response only caused by light stimuli.

***C*** *(types of control group)*: Adults with normal hearing.

***O*** *(types of outcome measures)*: PLR parameters and their relationships (correlations) with hearing status.

In addition, the following selection criteria were also imposed on all three sections of the review process.

- No animal studies.
- The language of the study was restricted to English only.
- Studies were published in a peer-reviewed scientific journal.
- Only quantitative studies were included; qualitative studies and case reports were excluded.

### Search strategy

Potential studies were identified by searching electronic databases and reviewing the reference lists of papers. Five electronic databases were selected to perform the search: PubMed, Embase, Cinahl, PsycINFO and Cochrane. The search result was last updated on 30^th^ October 2014 and there was no limitation with regard to the year of publication.

To perform the search, we first identified *Hearing Impairment*, *Pupil Light Reflex* and *Parasympathetic Nervous System* as the three key search terms in accordance to the objective of this review (Fig 3). Then for each database, we developed search strings for these key search terms individually by connecting keywords with the Boolean operator “OR” (see #1 to #3 in the sample search of PubMed shown in Table 1). We later paired the key search terms in correspondence to the three sections of the review, with Boolean operator “AND” used for the connection (#4 to #6 in Table 1). After that, the results from previous steps were combined to sum up a total number of items found from this database. Finally, a filter was applied to rule out all non-human studies. The search strategies for different databases were modified to meet their own standards (see S2 Appendix).

### Study selection

The retrieved publications from the database search and reference list check were imported into EndNote (version X7.1, Thomson Reuters). The following steps were performed in EndNote to refine the results: 1) we omitted duplicates from the retrieved publications; 2) we omitted the papers that were not written in English; 3) we omitted the results for which no abstract was available.

The refined results were exported with the identification number, title, and abstract (no authors were listed) for each study. Three reviewers (YW, SK, AZ) independently screened the results during the first screening stage. Three response options (“NO”, “YES” or “MAYBE”) were used to decide whether to include a study in the next stage. Reviewers also categorized each study into the three sections. To ensure the reliability of selection, any disagreement between the reviewers was resolved by discussing the abstract and full text of the paper if required. After the first screening, the publications included were inspected closely again by the three reviewers based on the full text, and the eligibility criteria of each section. Publications that survived this second screening were moved to the data extraction stage.

### Data extraction and presentation

We performed data extraction for each of the three sections separately (Fig 3). To this end, reviewer YW developed five draft sheets according to the eligibility criteria, which were reviewed by SK, AZ, GN and TL. YW extracted data from the included studies. Any disagreement among the reviewers regarding the draft sheets or extracted data was resolved by discussion and consensus. Extracted variables for each section were grouped into one of three categories: 1) Information about the nature of the participants, 2) Information about the intervention method and 3) Information about the outcome measure.

Finally, we formulated a hypothesis about the correlation between the two key search terms, as well as the direction (positive, negative or no correlation) of the hypothesized correlation. If the result of the outcome measure was in line with the hypothesis, a “+” sign was added to the data extraction sheet. If the result showed correlation in the opposite direction, then a “-” sign was given. Absent correlations were coded with “0”.

The extraction strategy of PLR and PNS dysfunction articles was as follows:

1. Information about the nature of the participants:
  - Type of PNS dysfunction
  - Number of participants for each group
  - Gender
  - Age
2. Information about the intervention method:
  - PLR measurement evaluating the PNS dysfunction of different groups.
  - Parasympathetic measurements other than PLR
3. Information about the outcome measure:
  - PLR parameters (e.g. absolute constriction amplitude, maximum constriction velocity)
  - Sensitivity of PLR parameters, i.e. whether a certain parameter showed a significant difference between groups
  - Whether a study fulfilled the hypothesis: *If a given condition is related to PNS dysfunction*, *then patients with this condition exhibit an attenuated PLR relative to healthy controls*. *In sub-groups of patients with diagnosed different levels of severity of the condition*, *more severe levels are associated with more attenuated PLR*.
  - Correlation between PLR parameters and other PNS measurement methods

## Results

### Study selection

In total, 2046 records were identified from the search of the five electronic databases. After removing duplicates, excluding records in languages other than English and discarding those records without any abstract, 1366 records remained for the first screening. We discarded 1219 records based on their abstract and title, leaving 147 records. Among those records, 138 belonged to PLR *AND* PNS dysfunction, 9 belonged to hearing impairment *AND* PNS. We failed to find any eligible studies about PLR *AND* hearing impairment.

**Fig 4:**
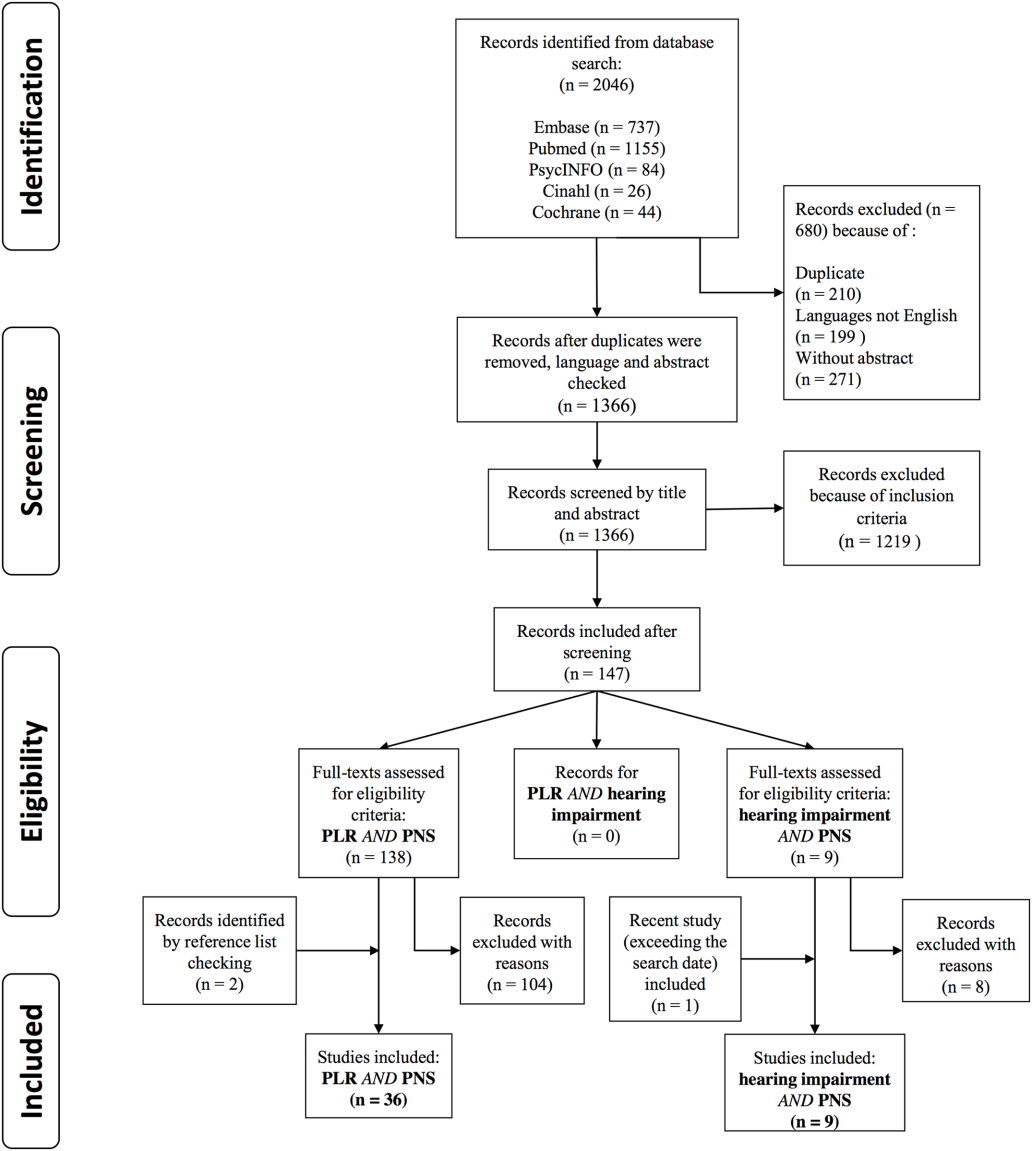
PRISMA flow diagram showing process for filtering search results and selecting studies for inclusion.

The full-texts of the 147 included articles were then reviewed independently by three researchers. This was done for each section according to their inclusion criteria, and this process was considered as the second screening. Seven of the 147 articles were review papers. These papers were not promoted to the data extraction stage but their reference lists were checked and they were used to extend the background knowledge. There were two studies sharing the same data, and we ruled out the one published earlier to avoid any bias. The second screening yielded 35 eligible papers in total for both sections, and by checking the reference list we found two more relevant papers. One additional study about hearing impairment *and* PNS was included, for which only the electronic copy of the paper was available online earlier than our cutoff date. We included it as there were so few papers in that section. Finally, there were 36 papers for PLR *and* PNS dysfunction, two for hearing impairment *and* PNS and none for hearing impairment *and* PLR (see Fig 4).

### Study characteristics

The characteristics of the studies described in the articles are presented separately for the two non-empty sections, and we generalized the information according to their relevance to our core objectives. The complete information of the study characteristics can be found in the appendix S3 Appendix.

**1. PLR and PNS Dysfunction**

Six parameters were selected as indices to assess PNS activity. These parameters are: 1) maximum constriction velocity (MCV), (the maximum slope of the constriction); 2) maximum constriction acceleration (MCA), (the maximum value of the second derivative of constriction); 3) absolute constriction amplitude (ACA), (the amplitude difference between baseline and minimum pupil diameter of the constriction); 4) relative constriction amplitude (RCA), (the ratio of ACA to baseline pupil diameter (BPD), expressed as a percentage); 5) latency, (the time between light stimulus onset and constriction onset); 6) constriction time, (the time from the end of latency period to the moment of maximum constriction). Specific values can be found in data extraction tables in the appendix. The data were first extracted by YW, then SK, AZ, TL, GN each randomly selected one study to extract the data and to compare their result with YW’s original result in order to check the data entries.

Studies investigating each different health condition were grouped together. For each study and each available PLR parameter, we noted whether statistically significant differences were found between the patient group and healthy controls or between different patient groups. Note that due to differences in test designs and light stimuli, it is impossible to directly compare the absolute values of PLR parameters between studies.

#### Parkinson’s Disease

Parkinson’s Disease (PD) is a degenerative disease of the nervous system with motor dysfunction. It is associated with PNS dysfunction, mainly due to an ACh and dopamine imbalance caused by dopamine deficiency [29]. Pupil abnormality was also described early on in PD research [30]. Four studies tested parasympathetic dysfunction in PD patients by PLR measurement [3, 4, 31, 32]. They all had age-matched groups of patients and healthy controls. Three out of four studies had at least one PLR parameter showing an attenuated PLR for PD patients in comparison with the normal controls (see Table 2). The fourth study [32] only used constriction velocity as the PLR parameter. The main purpose of that study was to identify relationships between olfaction and dysautonomia in PD, but the comparison between normal control and PD patients failed to find any difference in PLR. In contrast, ACA and Latency were sensitive to PD in the other three studies.

**Table 2:** Summary of the studies comparing PLR parameters between PD patients and normal controls.

PLR parameters	Studies including this PLR parameter	Result
MCV	[3, 4]	+ 0
MCA	[3, 4]	+ +
ACA	[3, 4, 31]	+ + +
RCA	[4]	+
Latency	[3, 4, 31]	+ + +
Constriction time	[4, 31]	0 +
Constriction velocity	[32]	0

Note: “+” indicates a result in agreement with the hypothesis of the present paper, “0” means that no statistically significant effect was found.

Giza et al. [3]

Fotiou et al. [4]

Micieli et al. [31]

Kang et al. [32]

#### Alzheimer’s Disease

Patients with Alzheimer’s disease usually suffer from reduced synthesis of ACh, which is the main neurotransmitter of the PNS. We found four eligible studies which examined the validity of the PLR as a measure of cholinergic (parasympathetic) dysfunction [4, 33, 34, 35]. Alzheimer’s disease patients were expected to have a reduced PLR in comparison with healthy controls. One recent study [35] did not show any differences between the Alzheimer’s disease and control groups for the four PLR parameters assessed. The remaining three studies all found at least one parameter which showed a group difference (see Table 3).

**Table 3:** Summary of the studies asessesing PLR parameters in Alzheimer’s disease patients and normal controls.

PLR parameters	Studies including this PLR parameter	Result
MCV	[4]	+
MCA	[4]	+
ACA	[4, 34, 35]	+ 0 0
RCA	[4, 33, 35]	+ + 0
Latency	[4, 33, 34, 35]	+ 0 0 0
Constriction time	[33, 34]	+ +

Note: “+” indicates a result in agreement with the hypothesis of the present paper, “0” means no statistically significant effect was found.

Fotiou et al. [4]

Tales et al. [33]

Fotiou et al. [34]

Bittner et al. [35]

#### Multiple System Atrophy

Multiple system atrophy is a degenerative neurological disorder with symptoms that are similar to, but usually more severe as those of PD [36]. Micieli et al. [37] measured PLR in multiple system atrophy patients, PD patients and healthy controls. They observed longer latency and constriction times and lower ACAs in multiple system atrophy patients in comparison with PD patients and controls. The result was believed to reflect a PNS imbalance in multiple system atrophy patients, and PLR was proposed as a potential tool for early diagnosis of multiple system atrophy.

#### Aging

The alteration of ANS function due to aging is well recognized [11, 38]. This alteration is usually characterized by a reduction of PNS tone in many parts of the body [39]. Two relevant studies used PLR to evaluate the PNS function across groups with different ages (see Table 4). Pozzessere et al. [40] divided their participants into four different groups according to age, and ACA and RCA were found to be larger in younger groups. MCV was only significantly different when a comparison was made between the youngest group and the oldest group. Bitsios et al. [8] found a smaller ACA and MCV in elderly participants regardless of light intensity. PLR latency of elderly participants was longer when compared with the young participants in a high-intensity condition only.

**Table 4:** Summary of the studies about the relationship between age and PLR parameters.

PLR parameters	Studies including this PLR parameter	Result
MCV	[8, 40]	+ + (only when youngest compared to oldest)
MCA	N/A	
ACA	[8, 40]	+ +
RCA	[40]	+
Latency	[8, 40]	+ 0
Constriction time	[40]	0

Note: “+” indicates a result in agreement with the hypothesis of the present paper, “0” means no statistically significant effect was found.

Bitsios et al. [8]

Pozzessere et al. [40]

#### Diabetes Mellitus

Autonomic neuropathy is a common disabling complication of Diabetes Mellitus (DM), and monitoring autonomic activity could help to identify the early onset of DM [41]. Pupillary defects are prevalent in patients with DM type 1 and type 2. The typical clinical signs can be summarized as fairly small and sluggish pupils, which are mainly due to the combined effect of iris damage and impairment of peripheral nerves [2]. PLR has been widely used to differentiate diabetic patients from normal controls. Eight of the nine included studies in DM patients measured PLR latency (see Table 5). All except one of these found longer constriction latency in DM patients. ACA was also found to differ between DM patients and controls in four out of six studies. For borderline DM (B-DM) and early-stage DM (E-DM), RCA was smaller in the B-DM group in comparison with the control and E-DM groups, and there were significant differences of MCV and ACA between E-DM and control groups Kuroda et al. [42].

**Table 5:** Summary of studies about the PLR in DM patients and normal controls.

PLR parameters	Studies including this PLR parameter	Result
MCV	[42, 43, 44, 45, 46, 47, 48]	+ (only E-DM vs control) + (only Diabetic Neuropathy (DNP) vs control) + + 0 0 + (DNP vs non-DNP)
MCA	N/A	
ACA	[42, 43, 44, 45, 46, 47, 48]	+ (only DNP vs control) + (only E-DM vs control) + + + 0 + (DNP vs non-DNP)
RCA	[42, 43, 44, 47]	+ (only B-DM vs control) 0 0 0
Latency	[5, 42, 43, 45, 46, 47, 49, 50]	+ (only DNP vs control) 0 + + + + + +
Constriction time	[45, 47]	0 0

Note: “+” indicates a result in agreement with the hypothesis of the present paper, “0” means no statistically significant effect was found.

Lanting et al. [5]

Kuroda et al. [42]

Zangemeister et al. [43]

Dutsch et al. [44]

Ferrari et al. [45]

Pfeifer et al. [46]

Levy et al. [47]

Yuan et al. [48]

de Vos et al. [49]

Pfeifer et al. [50]

#### Obesity

Previous studies suggest that obesity is usually associated with a decrease in cardiovascular PNS activity [51, 52]. Piha et al. [53] measured ANS activity in sets of identical twins in which one twin showed moderate obesity and the other normal weight. PLR measurements showed no difference between the obese and non-obese group on any parameters (see Table 6). Similarly, Baum et al. [54] failed to find any PNS-related parameter (including PLR) that differed between obese and normal-weight groups. Overall, PLR parameters do not appear likely to be sensitive to reduced PNS activity in obesity.

**Table 6:** Summary of the studies about the PLR in obese groups and non-obese controls.

PLR parameters	Studies including this PLR parameter	Result
MCV	[53, 54]	0 0
MCA	N/A	
ACA	[53, 54]	0 0
RCA	[53]	0
Latency	[54]	0
constriction time	[53]	0

Note: “+” indicates a result in agreement with the hypothesis of the present paper, “0” means no statistically significant effect was found.

Piha et al. [53]

Baum et al. [54]

#### Schizophrenia

Autonomic abnormalities are frequently reported in patients with Schizoprenia [55], and pupillary signs have a long history of use in the diagnosis of psychiatric conditions. Bumke [56] and Westphal [57] observed pupillary abnormalities in schizophrenic patients a century ago. Hakerem et al. [58] recorded the PLR in schizophrenia patients, having divided the patients into acute and chronic groups, and compared the data with a healthy control group. Of the four PLR parameters adopted, constriction time was found to diff between the patient groups (both chronic and acute groups) and the normal controls (see Table 7). Bar et al. [6] investigated whether cardiovascular autonomic dysregulation in schizophrenia patients might be reflected in the PLR. Only RCA differed between patients and controls.

**Table 7:** Summary of the studies about the influence of schizophrenia on PLR parameters.

PLR parameters	Studies including this PLR parameter	Result
MCV	[58]	0
MCA	N/A	
ACA	[58]	0
RCA	[6]	+
Latency	[6]	0
Constriction time	[58]	+
Flatness of curve	[58]	0

Note: “+” indicates a result in agreement with the hypothesis of the present paper, “0” means no statistically significant effect was found.

Bar et al. [6]

Hakerem et al. [58]

#### Migraine

Migraine is a common chronic debilitating neurological disease that causes severe headache. It usually involves some ANS symptoms like nausea, vomiting or diarrhea [59], and there is evidence suggesting the presence of PNS dysfunction during the onset of the disease [60, 61]. Three studies were identified from our search, and ACA and latency have been assessed in these studies (see Table 8). One study [62] found a significantly lower ACA in patients within two days of an attack, and one [63] observed a prolonged latency in migraine patients. MCV and constriction time were only examined in one of the three studies, but both were found to be sensitive to migraine. To summarize, each of these studies found at least one PLR parameter sensitive to migraine, but no strong consensus is apparent so far.

**Table 8:** Summary of the results of the studies about the relationship between migraine patients and normal controls when comparing with PLR parameters.

PLR parameters	Studies including this PLR parameter	Result
MCA	N/A	
ACA	[62, 63, 64]	0 + 0
RCA	N/A	
Latency	[62, 63, 64]	0 0 +
Constriction time	[62]	+
Constriction velocity (ACA/Constriction time)	[63]	+

Note: “+” indicates a result in agreement with the hypothesis of the present paper, “0” means no statistically significant effect was found.

Mylius et al. [62]

Micieli et al. [63]

Harle et al. [64]

#### Cluster Headache

A study by Drummond [65] suggested PNS hyperactivity during the active phase of headache. On the other hand,Ofte et al. [66] found decreased MCV, RCA, average constriction velocity, and prolonged latency in cluster headache patients during the remission phase of the headache. This result indicates a reduced PNS tone for cluster headache, and seems to be contradictory to the result of Drummond. Ofte and co-workers concluded that their result may indicate a chronic down-regulation of PNS to avoid further attacks.

#### Arthritis

Abnormality of ANS functioning is well recognized in arthritis disease [67]. Studies have suggested an altered cardiovascular PNS dysfunction in rheumatoid arthritis patients [68, 69]. We found two studies with PLR fulfilling the inclusion criteria (see Table 9). Perry et al. [70] tested both PLR and cardiovascular function in patients with inflammatory arthritis, and they found a significantly lower MCV and RCA in patients compared to healthy controls. They suggested that this reflects a decreased PNS tone as it was confirmed by their heart rate variability (HRV) data. As one of the most widespread way to evaluate PNS function, heart rate variability is derived from the electrocardiogram, and a reduced high frequency component of heart rate variability is usually linked to a reduction of PNS activity [71]. Barendregt et al. [72] found that latency and time to MCV were prolonged in rheumatoid arthritis patients with ocular dryness compared to patients without ocular dryness and normal controls.

**Table 9:** Summary of the studies about the influence of rheumatoid arthritis on the PLR.

PLR parameters	Studies including this PLR parameter	Result
MCV	[70]	+
MCA	N/A	
ACA	N/A	
RCA	[70]	+
Latency	[72]	+
Average Constriction Velocity	[70]	+
Constriction time	[72]	+

Note: “+” indicates a result in agreement with the hypothesis of the present paper, “0” means no statistically significant effect was found.

Perry et al. [70]

Barendregt et al. [72]

#### Meniere’s Disease

Meniere’s disease is a disorder of the inner ear that is known to cause hearing-related problems like tinnitus and hearing loss. Research by Yamada et al. [73] suggested that patients present a PNS hypo-function during the interval stage (i.e. between attacks), as reflected in the high frequency component of HRV. Inoue and Uemura [74] observed that MCV was lower on the affected side of Meniere’s diseasepatients than in normal controls. They concluded that this observation indicated PNS dysfunction. This research comes close to making a connection between hearing impairment and PLR, but unfortunately the authors did not report the hearing characteristics of the patient group.

#### Multiple Sclerosis

Multiple sclerosis is an inflammatory autoimmune disorder that can involve disseminated lesions of the central nervous system and lead to autonomic disturbances. Evidence from cardiovascular measurements suggests that PNS dysfunction is common among multiple sclerosis patients [75, 76]. We identified one study which used PLR to study the autonomic function in multiple sclerosis. Pozzessere et al. [77] categorized participants according to their baseline pupil size, so the comparisons were made between healthy controls and patients with various baseline pupil sizes. MCV and RCA were smaller in multiple sclerosis patients than in the control group, except for those having baseline pupil sizes larger than 6.95 mm.

#### Heart Failure

Heart failure is usually associated with decreased PNS function, as reflected by HRV measures [78]. One PLR study [79] was identified, and it demonstrated significantly lower MCV together with longer latency and constriction time in heart failure patients. The authors believed this result indicated a cholinergic deficiency in the cardiovascular system and a reduced PNS tone for heart failure patients.

#### Familial Dysautonomia

Familial dysautonomia is a rare genetic disease that affects development of the sensory and autonomic nervous systems, including part of the PNS neurons. Dutsch et al. [80] tested PLR in familial dysautonomia patients and normal controls, and they found all the tested PNS-related parameters (MCV, ACA and RCA) were significantly reduced in familial dysautonomia patients.

#### Generalized Autonomic Neuropathy

We identified two studies whose patient groups exhibited a variety of autonomic neuropathies (many of these neuropathies were already mentioned above) rather than one specific disease. Bremner and Smith [81] recorded PLR in 150 patients with symptomatic autonomic neuropathies. The diagnoses were made clinically and included multiple system atrophy, pure autonomic failure, DM and amyloidosis. PLR findings suggested patients with DM or amyloidosis had significantly attenuated ACA in comparison with normal controls, and patients with multiple system atrophy or pure autonomic failure. Similarly, in Muppidi et al. [82] autonomic dysfunctions were determined by the Composite Autonomic Severity Score (CASS). The score was derived from the autonomic reflex screening including HRV and blood pressure. When comparing high-CASS patients with normal controls and normal-CASS patients, it was found that MCV, ACA, and RCA were lower in the high-CASS group. This result indicated a PNS deficit in high-CASS patients.

**2. Hearing Impariment and Parasympathetic Nervous System**

Ten results were promoted after the first screening for this section. During the second screening, eight of them were discarded due either to a lack of a proper measurement of hearing status or a failure to evaluate relations between hearing impairment and parameters evaluating PNS activity.

Hasson et al. [83] studied correlations between hearing problems and parasympathetic markers of cardiovascular activity as reflected by HRV. Forty-seven musicians from orchestras participated. Hearing problems were assessed by a questionnaire, including items about hearing loss, tinnitus and hyperacusis. Hearing problems were found to be negatively correlated with the high-frequency (HF) component of HRV, which is known to be a valid indicator of PNS activity [71]. The low-frequency HRV power, which reflects the balance between PNS and SNS activity, was also found to be negatively correlated with hearing problems after age and gender adjustment. Given that PNS activity is responsible for stimulation of ‘rest and digest’ functions, PNS activity could play a protective role in helping to recover from hearing-related stress. Therefore, the authors further suggested that people with hearing problems might have a reduced ability to ‘unwind’ from the stress or difficulties caused by their hearing problems.

While Hasson et al. [83] mainly focused on the chronic effect of hearing problems on the PNS, Mackersie et al. [27] investigated how PNS activity differs between normal-hearing and hearing-impaired listeners during sentence recognition tasks (i.e. acute effects). The study included 18 adults with sensorineural hearing loss and 15 normally-hearing adults, as determined by pure-tone audiometry. Participants were required to recognize sentences in noise at a range of signal to noise ratios, and their electrocardiograph (ECGs) were recorded during the test. The HF component of HRV in participants with hearing loss was lower than in normally-hearing participants at the lower (more difficult) signal to noise ratios, and it also showed a clear tendency of decreasing under the more difficult listening conditions for hearing impairment participants, but not for the normally-hearing participants. On the other hand, participants with hearing loss showed increased skin conductance reactivity to noise, which indicates the mere presence of noise triggers a greater sympathetic activation in the hearing-impaired. The authors suggested that the result could be interpreted as participants with hearing loss experiencing more stress-induced autonomic activation and using more cognitive resources in general during the tasks.

## Discussion

In total, 2046 articles were found from our primary systematic search, and 38 of them met the inclusion criteria and were included as relevant studies after the selection procedure. Among them, 36 addressed the use of PLR to measure PNS activity and the other two were about hearing impairment *and* PNS. No studies were identified considering the association between hearing impairment *and* PLR.

### 1. PLR and PNS Dysfunction

The first main purpose of this review was to assess the sensitivity of PLR as a method to evaluate PNS dysfunction, since we found that there is no up-to-date systematic review available to generalize the research in this field. Across the various types of PNS dysfunction in the survey, 30 out of 36 studies clearly stated their findings of PNS dysfunction or decreased PNS tone in the patient group on the basis of PLR measurements in their results or discussion sections. Another 2 studies [58, 63] failed to mention PNS dysfunction directly, although they observed a reduced PLR in their patient groups, and the remaining 4 studies [32, 53, 54, 84] failed to observe any relation between PNS abnormality and PLR test results. Taken together, these results could reasonably be interpreted as evidence for the sensitivity of PLR as a testing tool for the evaluation of PNS dysfunction. None of the studies showed an opposite effect: better PNS function in groups of patients with cholinergic deficits.

Different parameters of the PLR were adopted in different studies. Whereas the PLR taken as a whole is governed by both SNS and PNS, the constriction phase is mainly controlled by PNS, so the parameters relating to pupil constriction may be considered as PNS-related PLR parameters. Bearing in mind that all parameters were derived from the same raw pupillometry data recording, it is still worth knowing which PLR parameters are the best indicators of PNS dysfunction. Table 10 shows the number of occurrences of each PLR parameter in all the studies in the PLR *and* PNS dysfunction survey. Although latency was used most often, MCV seems to possess a higher likelihood of detecting PNS dysfunction. ACA and RCA also appear promising in this regard, whereas MCA and Time to MCV are insufficiently studied to draw any conclusions.

**Table 10:** Counts of PLR constriction parameters used in all the 36 PLR+PNS studies.

	Latency	ACA	MCV	RCA	Constriction time	MCA	Time to MCV
Total N	28	24	21	15	12	4	2
N showing difference	16	18	18	11	6	3	2
% showing difference	57	75	86	73	50	75	100

*Total N*, how many studies used the given parameter; *N showing difference*, how many studies found a significant difference in the given parameter between patient and control groups; *% showing difference*, = (N showing difference) / (Total N) x 100.

The nature of PNS-related diseases is crucial as it is the determinant of PNS dysfunction. Therefore, we have categorized studies according to different types of diseases to evaluate the effectiveness of PLR parameters within the same disease or PNS dysfunction. Most diseases found at least one PLR parameter to show decreased PNS tone in the patient group. Nevertheless, there are 4 exceptions, and two of them were studies about obesity [53, 54]. Although there are researches suggesting obesity is associated with decreased PNS activity [51, 52], it is possible that PNS dysfunction in obesity is less severe compared with other diseases. By contrast, diabetes mellitus, a disease with pronounced PNS involvement, was the object of investigation by PLR in 9 studies in our survey. Latency, ACA and MCV were all sensitive parameters to differentiate diabetes mellitus patients from healthy controls. Alzheimer’s disease, Parkinson’s disease, multiple system atrophy and aging could be grouped together as neurological degenerative conditions. For these conditions, MCV, ACA and RCA generally demonstrated sensitivity to reduced PNS activity caused by cholinergic deficiency.

Latency (the time gap between light stimulus onset and start point of constriction) was found to be the most often-used PLR parameter, but not the most sensitive one to PNS dysfunction. It is worthwhile to pursue this point further. Most studies that adopted Latency claimed that this parameter was an uncontaminated indicator of PNS activity, and that PNS dysfunction usually resulted in a longer Latency. Many of these studies cited the paper of Pfeifer and co-workers [46] that described Latency as a PNS indicator. In that paper, multiple SNS and PNS stimulation and blockade drops were administered to diabetic patients and healthy controls, and prolonged latency as a sign of PNS impairment was part of the authors’ interpretation of their test results. However, we could not find any in-depth explanation which clearly establishes a basis for the latency being an indicator of parasympathetic functioning. According to Loewenfeld and Lowenstein [2], PLR latency is composed of two different components, “the irreducibly minimal latent period built into the motor system of the iris”, and “a variable additional delay”. The minimal latent period in humans is about 180 to 200 ms [2]. Certainly lower light intensity [85] and older age [86] can prolong the latency, but regardless of the light intensity and age, the additional delay is “almost exclusive to retinal output and is only slightly modified by central nervous inhibition” [87]. Thus, it may be open to further debate whether Latency is likely to be a reliable PNS indicator. We suggest that subsequent research needs to take this into consideration when using Latency as a PLR parameter to evaluate PNS activity.

MCV, ACA and RCA, on the contrary, are under direct control of the PNS system, and a reduced light reflex could be explained by reduction of activity in preganglionic parasympathetic fibers and ciliary ganglion neurons [2]. One may argue that MCV and ACA are likely to be correlated to the baseline pupil diameter, which reflects the balance between PNS and SNS, so that they might not be pure indicators of PNS activity. However, evidence suggests that MCV is independent of the baseline pupil diameter in healthy subjects [79, 88]. RCA is calculated as the ratio of ACA to baseline pupil diameter, so its dependency on baseline pupil diameter is eliminated [79], which probably makes it more robust than ACA as an indicator of PNS activity. Most of the PLR studies measured multiple PLR parameters simultaneously, but the interdependence of these parameters is rarely considered. For example, Bremner [88] found a striking and tight linear correlation between MCV and ACA. However, we did not find any study suggesting a correlation between MCV and RCA. As a result, MCV and RCA might be the best PLR parameters to detect PNS dysfunction.

Another perspective on the utility of PLR for detecting PNS dysfunction is to examine the correlation of PLR parameters with other established PNS measurement methods. For example, HRV parameters were significantly correlated (R = 0.56 for the HF component of HRV and ACA, R = 0.83 for the HF component of HRV and MCA, R = 0.62 for the HF component of HRV and MCV) with PLR parameters in healthy athletes during exercise [89]. Four studies included in this review also conducted cardiovascular measurements, and the cardiovascular results showed reduced PNS tone in line with their pupillometric findings [6, 47, 50, 70]. While these studies appear unanimous, it would be premature to conclude on the basis of so relatively little evidence.

Although the technical demands of PLR measurement are relatively straightforward, it is also worth mentioning that PLR is a test that might be influenced by many factors other than those one wishes to study. The characteristics of the light stimuli [90], gender [91], smoking [92], alcoholism [93] and even the color of iris [94] could influence the test result. Many studies in our review failed to report these factors. Since such confounding factors might introduce bias and extra variance into experimental results, they should preferably be included as covariates or exclusion criteria in PLR studies. Such confounding factors might make it impossible to compare the absolute magnitude of the PLR parameters across different studies.

Moreover, effort has been made to quantify the PNS and SNS input in PLR by constructing mathematic models. Usui and Hirata [95] and Yamaji et al. [96] proposed a modeling method called ‘pupillary muscle plant’, where tension of the sphincter and dilator muscle, as well as the SNS and PNS contribution were considered as the input of the model system. By inverse dynamic modeling, they were able to estimate the PNS contribution based on PLR response, and the whole model was validated by experimental testing. Fan and Yao [97] built a similar model where PNS activity was considered as part of the input. They extracted the PNS contribution from experimental data and found a higher PNS activity in females than in males, which is in line with previous findings based on cardiovascular measurements [98].

### 2. Hearing Impariment and Parasympathetic Nervous System

The second purpose of this systematic review was to investigate the relationship between hearing impairment and PNS. Only two relevant studies [17, 27] were identified. Both studies used the high-frequency (HF) component of HRV to evaluate the PNS activity, and they both found a reduced HF component of HRV in participants with hearing impairment compared with normally hearing participants. However, the reduced HF HRV in Hasson et al. [17] is most likely due to the long term effects of their respondents’ hearing problems, while the HRV finding of Mackersie et al. [27] is more likely caused by short-term stress during the speech recognition task. Thus it appears possible that PNS plays an important role when dealing with both short-term and long-term stress in hearing-impaired listeners. Hasson et al. [17] suggested that the reduced PNS activity in people with hearing impairment might indicate a worse ability to ‘unwind’ or recover from the stress related to their long lasting hearing problems.

However, both papers failed to clarify any exclusion critieria for their participants regarding any autonomic dysfunction conditions, as we described previously. It should be stressed here that hearing impairment might have a link to some of these conditions. For example, evidence suggests that hearing impairment is about twice as common in adults with diabetes compared to healthy controls [99], and the higher prevalance of hearing loss in Alzheimer’s patients is also frequently reported [100]. Thus, bias might be introduced when interpreting the relationship between hearing impairment and parasympathetic activity. Furthermore, the fact that only two studies were identified from our systematic search leaves too little evidence to draw any conclusions about our second goal. Nevertheless, we believe the lack of evidence actually demonstrates the need for future studies to bridge the gap between hearing impairment and parasympathetic activity. For any such future studies, it will be important to exclude subjects diagnosed with PNS-related conditions, in order to avoid possible confounds.

### 3. PLR and Hearing Impairment

Our search did not identify any studies connecting the PLR and hearing impairment. This is another major outcome of this study. Apparently, the method is still unknown in the field of Audiology, despite the fact that pupillometry equipment and techniques are already in use in audiological research to assess sympathetic activity or listening effort [101, 102, 103], and PLR can be measured with the same setup. The typical use of pupillometry in audiological research (measurement of pupil dilation as an indicator of the allocation of cognitive resources) has hitherto assumed that the pupil dilation response is solely affected by SNS activity, whereas in fact there may be some contamination by PNS activity [104]. For this reason, the study of PLR could be valuable for further refining experimental techniques, and by promoting the disentanglement of SNS and PNS effects. Thus, future studies might adopt PLR as a testing tool to evaluate the possible existence of PNS dysfunction in people with hearing impairment.

### Limitations

1. There is a risk of bias when only including studies mentioning PNS or similar terms in their title or abstract, because if a study failed to find any difference from PNS-related PLR parameters, there would be a lower probability for it being mentioned in the title or abstract.
2. In order for a given disease to be included, it had to be ‘well-known’ that it is related to PNS dysfunction. To determine this relationship, we searched ‘PNS’ and the name of each condition via electronic database. Some studies were thus excluded because we could not find any direct evidence suggesting its association to PNS dysfunction. This procedure is not absolutely objective, and may have introduced bias to our results.
3. Comparison of PLR parameters: In general, there are two basic elements needed to complete a PLR test: light stimuli and a device to record pupil size. The characteristics of the PLR are highly dependent on the type of the light stimulus. Intensity, duration, waveform and frequency of the light all contribute to the reflex pattern [2]. However, researchers habitually apply different types of light stimuli in their PLR studies. Therefore, the results from different studies are usually not directly comparable. This might generate some unreliability in our result and discussion, as we grouped results from studies according to the type of disease and not the stimulus characteristics.
4. We did not perform any quality assessment on the included studies due to the rather broad topic covered in this review. This fact may generate risk and bias to the review as the quality of studies varies from one to another.
5. This review only focused on the evaluation of chronic PNS dysfunction using PLR measurement. However, PLR is also used extensively in the evaluation of task-induced PNS activity due to short term stress exposure. For example, there are studies recording PLR during numerical counting [105] in order to investigate the impact of cognitive processing on PLR. Threat of electric shock [106] and cold-pressor tests [107] have been conducted while simultaneously measuring PLR to study the modulation of the PLR by acute stress. Future research may need to take this into consideration in order to gain a better understanding of PLR.
6. Only two papers discussing the relationship between hearing impairment and PNS were identified from our systematic search. This lack of evidence makes it hard for us to draw any conclusion on our second goal.

## Conclusions

The major outcomes of this systematic review are:

1. This is the first systematic review of the evidence concerning the sensitivity of PLR as a method to evaluate parasympathetic dysfunction. The relationships between PLR and PNS dysfunction were evaluated by reviewing the sensitivity of PLR as a testing method to differences between patients with PNS-related dysfunctions and healthy controls. Most of the included studies found at least one PLR parameter to be significantly different between the patient group and control group.
2. The PLR parameters most likely to be sensitive to PNS dysfunction appear to be the Maximum Constriction Velocity (MCV) and Relative Constriction Amplitude (RCA).
3. We only found two studies investigating the PNS activity in people with hearing impairment. The evidence base is thus weak, but points to reduced PNS activity in people with hearing impairment.
4. We found no studies in the existing literature connecting PLR and hearing impairment. We believe this systematic review can help to bridge the gap between PLR and hearing impairment and may show a new direction for future research.

## Supporting Information

S1 Appendix PRISMA Checklist. (DOC)

S2 Appendix Systematic search for five databases. (DOC)

S3 Appendix Table for PLR *AND* Parasympathetic functions. (DOCX)
