# Supplementary material for: Parasympathetic Nervous System Dysfunction, as Identified by Pupil Light Reflex, and Its Possible Connection to Hearing Impairment
Source: PLoS One. 2016 Apr 18;11(4):e0153566. doi: 10.1371/journal.pone.0153566 (PMC4835104; doi:10.1371/journal.pone.0153566)
Supplement: S2 Appendix — (DOC) [file pone.0153566.s002.doc]

# PubMed:

**#1 Hearing impairment**

"Hearing"[Mesh:NoExp] OR "Hearing disorders"[Mesh] OR "Persons With Hearing Impairments"[Mesh] OR "hearing loss"[Mesh] OR Presbyacusis[tiab] OR Presbyacusia[tiab] OR Presbycusis[tiab] OR hypoacusis[tiab] OR Deaf*[tiab] OR (Hearing[tiab] AND (condition*[tiab] OR disabilities[tiab] OR disability*[tiab] OR disabled*[tiab] OR disorder*[tiab] OR handicap*[tiab] OR impair*[tiab] OR loss[tiab] OR Problem*[tiab]))

**#2 PLR (pupil light reflex)**

"Reflex, Pupillary"[Mesh] OR "Pupillary reflex"[tiab] OR "pupil reaction"[tiab] OR "pupil reflex"[tiab] OR "light reflex"[tiab] OR "pupillary reaction"[tiab] OR "pupillary reactivity"[tiab] OR "pupil reactivity"[tiab] OR "pupillary response"[tiab] OR "pupil response"[tiab] OR "dynamic pupil"[tiab] OR "flash response"[tiab]

**#3 Parasympathetic nervous system**

"Parasympathetic Nervous System"[Mesh] OR "Acetylcholine"[Mesh] OR "parasympathetic"[tiab] OR "cholinergic"[tiab] OR "Vagus Nerve"[tiab] OR "Nerves vagus"[tiab] OR "ciliary ganglion"[tiab] OR "ganglion ciliare"[tiab] OR "ganglion opticum"[tiab] OR "optic ganglion"[tiab]

**#4** NOT ("animals"[MeSH Terms] NOT "humans"[MeSH Terms])

# EMBASE.com:

#1 Hearing impairment

'hearing'/de OR 'hearing disorder'/exp OR 'hearing impairment'/exp OR 'hearing impaired person':ti,ab OR 'hearing impaired persons':ti,ab OR Presbyacusis:ti,ab OR Presbyacusia:ti,ab OR Presbycusis:ti,ab OR hypoacusis:ti,ab OR Deaf*:ti,ab OR (Hearing NEXT/1 (condition* OR disabilit* OR disabled OR disorder* OR handicap* OR impair* OR loss OR Problem*)):ti,ab

**#2 PLR (pupil light reflex)**

'pupil reflex'/exp OR ((Pupil* OR light OR flash) NEXT/1 (reflex* OR reaction* OR reactivit* OR respons*)):ti,ab OR 'dynamic pupil':ti,ab

**#3 Parasympathetic nervous system**

'cholinergic system'/exp OR 'acetylcholine'/exp OR 'parasympathetic':ti,ab OR 'cholinergic":ti,ab OR 'Vagus Nerve':ti,ab OR 'Nerves vagus':ti,ab OR 'ciliary ganglion':ti,ab OR 'ganglion ciliare':ti,ab OR 'ganglion opticum':ti,ab OR 'optic ganglion':ti,ab

**#4 human studies filter**

# PsycINFO

**#1 Hearing impairment**

DE "Hearing Disorders" OR DE "Deaf" OR DE "Deaf Blind" OR TI hearing OR TI Presbyacusis OR TI Presbyacusia OR TI Presbycusis OR TI hypoacusis OR TI Deaf* OR (TI Hearing AND (TI condition* OR TI disabilities OR TI disability* OR TI disabled* OR TI disorder* OR TI handicap* OR TI impair* OR TI loss OR TI Problem*)) OR AB hearing OR AB Presbyacusis OR AB Presbyacusia OR AB Presbycusis OR AB hypoacusis OR AB Deaf* OR (AB Hearing AND (AB condition* OR AB disabilities OR AB disability* OR AB disabled* OR AB disorder* OR AB handicap* OR AB impair* OR AB loss OR AB Problem*))

**#2 PLR (pupil light reflex)**

TI ("Pupillary reflex" OR "pupil reaction" OR "pupil reflex" OR "light reflex" OR "pupillary reaction" OR "pupillary reactivity" OR "pupil reactivity" OR "pupillary response" OR "pupil response" OR "dynamic pupil" OR "flash response") OR AB ("Pupillary reflex" OR "pupil reaction" OR "pupil reflex" OR "light reflex" OR "pupillary reaction" OR "pupillary reactivity" OR "pupil reactivity" OR "pupillary response" OR "pupil response" OR "dynamic pupil" OR "flash response")

**#3 Parasympathetic nervous system**

DE "Parasympathetic Nervous System" OR DE "Efferent Pathways" OR DE "Vagus Nerve" OR DE "Acetylcholine" OR TI ("parasympathetic" OR "cholinergic" OR "Vagus Nerve" OR "Nerves vagus" OR "ciliary ganglion" OR "ganglion ciliare" OR "ganglion opticum" OR "optic ganglion") OR AB ("parasympathetic" OR "cholinergic" OR "Vagus Nerve" OR "Nerves vagus" OR "ciliary ganglion" OR "ganglion ciliare" OR "ganglion opticum" OR "optic ganglion")

**#4 filter for human studies**

# Cinahl

**#1 Hearing impairment**

(MH "Hearing Disorders+") OR (MH "Deafness+") OR (MH "Hearing Loss, Partial+") OR (MH "Hearing+") OR (MH "Deaf-Blind Disorders+") OR TI hearing OR TI Presbyacusis OR TI Presbyacusia OR TI Presbycusis OR TI hypoacusis OR TI Deaf* OR (TI Hearing AND (TI condition* OR TI disabilities OR TI disability* OR TI disabled* OR TI disorder* OR TI handicap* OR TI impair* OR TI loss OR TI Problem*)) OR AB hearing OR AB Presbyacusis OR AB Presbyacusia OR AB Presbycusis OR AB hypoacusis OR AB Deaf* OR (AB Hearing AND (AB condition* OR AB disabilities OR AB disability* OR AB disabled* OR AB disorder* OR AB handicap* OR AB impair* OR AB loss OR AB Problem*))

**#2 PLR (pupil light reflex)**

TI ("Pupillary reflex" OR "pupil reaction" OR "pupil reflex" OR "light reflex" OR "pupillary reaction" OR "pupillary reactivity" OR "pupil reactivity" OR "pupillary response" OR "pupil response" OR "dynamic pupil" OR "flash response") OR AB ("Pupillary reflex" OR "pupil reaction" OR "pupil reflex" OR "light reflex" OR "pupillary reaction" OR "pupillary reactivity" OR "pupil reactivity" OR "pupillary response" OR "pupil response" OR "dynamic pupil" OR "flash response")

**#3 Parasympathetic nervous system**

(MH "Parasympathetic Nervous System+") OR (MH "Vagus Nerve") OR (MH "Acetylcholine") OR TI ("parasympathetic" OR "Efferent Pathways" OR "cholinergic" OR "Vagus Nerve" OR "Nerves vagus" OR "ciliary ganglion" OR "ganglion ciliare" OR "ganglion opticum" OR "optic ganglion") OR AB ("parasympathetic" OR "Efferent Pathways" OR "cholinergic" OR "Vagus Nerve" OR "Nerves vagus" OR "ciliary ganglion" OR "ganglion ciliare" OR "ganglion opticum" OR "optic ganglion")

# Cochrane Library

**#1 Hearing impairment**

"Hearing" OR Presbyacusis OR Presbyacusia OR Presbycusis OR hypoacusis OR Deaf*

**#2 PLR (pupil light reflex)**

"Pupillary reflex" OR "pupil reaction" OR "pupil reflex" OR "light reflex" OR "pupillary reaction" OR "pupillary reactivity" OR "pupil reactivity" OR "pupillary response" OR "pupil response" OR "dynamic pupil" OR "flash response"

**#3 Parasympathetic nervous system**

"Parasympathetic" OR "Acetylcholine" OR "cholinergic" OR "Vagus Nerve" OR "Nerves vagus" OR "ciliary ganglion" OR "ganglion ciliare" OR "ganglion opticum" OR "optic ganglion"
