# Supplementary material for: Parasympathetic Nervous System Dysfunction, as Identified by Pupil Light Reflex, and Its Possible Connection to Hearing Impairment
Source: PLoS One. 2016 Apr 18;11(4):e0153566. doi: 10.1371/journal.pone.0153566 (PMC4835104; doi:10.1371/journal.pone.0153566)
Supplement: S3 Appendix — (DOCX) [file pone.0153566.s003.docx]

S3 Appendix. Table for PLR *AND* Parasympathetic functions

| **Reference** | **Type of parasympat-hetic dysfunction** | **Nature of groups and group comparisons** | **Number of participants** | **Mean age**  **(yr)**  **(SD)** | **Descriptive data for PLR result on patient group and ANOVA/ANCOVA result for PLR in patient group and normal control group (if applicable)** | | | | | | | | | **Additional measurement of parasympathetic function** | **Assumption** |
| --- | --- | --- | --- | --- | --- | --- | --- | --- | --- | --- | --- | --- | --- | --- | --- |
|  |  |  |  |  | **MCV**  $\boldsymbol{mm/s}$  **(SD)** | **MCA** $\boldsymbol{mm/}\boldsymbol{s}^{\boldsymbol{2}}$  **(SD)** | **ACA**  $\boldsymbol{mm}$  **(SD)** | **RCA**  **%**  **(SD)** | **Latency**  $\boldsymbol{s}$  **(SD)** | **Constrict-ion time**  **s**  **(SD)** | | **Other PLR parameters** | |  |  |
| Giza et al. [17] | Parkinson’s disease (PD) (idiopathic) | PD patient  normal control  patient vs normal | 66 (44 males)  44 (20 males) | 64.1 (8.4)  age matched | 1.87 (0.46)  2.30 (0.43)  * | 16.61 (4.60)  21.83 (3.35)  *^ | 0.54 (0.16)  0.69 (0.13)  * | N/A | 0.252 (0.016)  0.236 (0.018)  * | N/A | | N/A | | N/A | + |
| Micieli et al. [28] | Parkinson’s disease (PD) | PD patient  normal control  patient vs normal | 23 (15 males)  26 (20 males) | 58.5 (9.9)  54.2 (11.3) | N/A | N/A | 1.35 (0.47)  1.53 (0.31)  * | N/A | 0.334 (0.060)  0.309  (0.026)  *^ | 0.461 (0.107)  0.411 (0.056)  * | | N/A | | N/A | + |
| Tales et al. [30] | Alzheimer’s disease (AD) | AD patients  older control  young control  AD vs older&young control | 12 (4 males)  12 (9 males)  12 (5 males) | 70 (7)  70 (6)  29 (5.6) | N/A | N/A | N/A | 0.221 (0.12)  0.316 (0.08)  0.333 (0.09)  * | 0.663 (0.37)  0.485 (0.05)  0.455 (0.03)  ns | N/A | | N/A | | N/A | +  only RCA was significantly different |
| Muppidi et al. [76] | Autonomic dysfunction, defined by CASS score.  Part of the high CASS patients were with diabetes, Parkinson | normal CASS score  high CASS score  normal control  high CASS vs normal CASS/normal | 28  26  79 | 41.7 (15.3)  65.1 (11.4)  41.3 (14.8) | 4.62 (0.73)  3.44 (1.02)  4.91 (0.73)  *^ | N/A | 1.61 (0.3)  1.03 (0.3)  1.61 (0.3)  * | 27.8 (5.1)  24.7 (6.4)  29.4 (4.6)  * | N/A | N/A | | N/A | | -QSART (Quantitative Sudomotor Axon Reflex Test)  ${HR}_{DB}$ range  -blood pressure  -HR during Valsalva maneuver  all of the measurements above contributed to the CASS score | + |
| Zangemeister et al. [39] | Diabetes mellitus (DM)  both type 1 and type 2 were included | Patients with two signs of diabetic neuropathy (DNP) as subgroup  All DM patients (including DNP group)  Normal control  DNP vs normal  all DM vs normal | 23  52 (31 males)  21 (11 males) | 59.7 (10.1)  46.7  46.2 (14.5) | 3.44 (1.28)  4.16 (1.22)  4.42 (0.93)  *  ns | N/A | 1.26 (0.45)  1.49 (0.41)  1.53 (0.34)  *  ns | 30.6 (9.02)  31.0 (8.59)  29.1 (7.64)  ns  ns | 0.28 (0.04)  0.27 (0.09)  0.25 (0.03)  *  ns | N/A | | N/A | | N/A | + |
| Lanting et al. [19] | Diabetes mellitus (DM) | DM patient  normal control  DM vs normal | 29 (14 males)  29 (missing gender) | 45.9 range (18-75)  40.0 range (18-76) | N/A | N/A | N/A | N/A | 0.236(SEM: 0.006)  0.211 (SEM: 0.004)  * | N/A | | N/A | | - thermal discrimination threshold  -vibratory perception threshold  -motor nerve donduction velocity  -H-M interval  thermal discrimination threshold showed a positive correlation with the latency | + |
| Bar et al. [20] | Paranoid schizophrenia | patient  normal control  patient vs normal | 28 (10 males)  28 (10 males) | 31 range (18-67)  32 range (18-54) | N/A | N/A | N/A | 28.0 (5.2)  24.6 (5.2)  * | 0.248 (0.016)  0.251 (0.019)  ns | N/A | | N/A | | -HRV  -blood pressure  -baroreflex sensitivity  all 3 measurements showed a reduced parasympathetic function in line with the PLR result | + |
| Mylius et al. [57] | Migraine headache | patient  normal control  patient within 48 hours of an attack as subgroup  all patient vs normal  48 hours vs normal | 42 (10 males)  42 (12 males)  10 | 27 (5.3) range (20-40)  26.4 (3.6) range (21-36)  N/A | 4.87 (0.7)  4.97 (0.9)  4.5 (0.8)  ns  * | N/A | 1.84 (0.3)  1.89 (0.3)  1.74 (0.3)  ns  * | N/A | 0.231 (0.02)  0.232 (0.02)  0.235 (0.02)  ns  ns | N/A | |  | | N/A | +  a parasympathetic dysfunction reflected by PLR could only valid within 48 hours of an attack |
| Ofte et al. [61] | Episodic Cluster Headache (CH) (during remission) | CH patient:  affected side  non-affected side  normal control  patient vs normal  affected side vs non-affected side | 30 (27 males)  30 (gender matched) | 50.2 (13.6)  age matched | 4.41 (1.2)  4.51 (1.17)  5.11 (0.63)  *  ns | N/A | (min diameter)  4.02 (1.21)  4.03 (1.15)  3.95 (0.72)  ns  ns | 28.52 (4.17)  29.24 (4.13)  32.70 (3.75)  *  ns | 0.24 (0.02)  0.24(0.02)  0.22 (0.01)  *  ns | N/A | | average constriction velocity  *  * | | N/A | + |
| [73]Keivanidou et al., 2010 | Heart failure (HF) patient | HF patient  normal control  patient vs normal | 16  16 | 67.5 (11.2)  age matched | 1.83 (0.18)  2.40 (0.24)  * | 24.60 (3.36)  14. 20 (0.27)  * | N/A | (minimum/baseline)  97.49 (1.29)  93.96 (4.11)  * | 0.249 (0.021)  0.233  (0.018)  * | N/A | |  | | N/A | + |
| de Vos et al. [45] | Diabetes mellitus (DM) | DM patients  normal control  patient vs normal | 18  18 | 45 (22-62)  39 (18-62) | N/A | N/A | N/A | N/A | 0.248 (0.025)  0.200  (0.020)  * | N/A | |  | | N/A | + |
| Micieli et al. [58] | Migraine | migraine patient  -pain side  -pain-free side  normal control  patient vs normal | 38 (24 males)  22 (gender matched) | 37.3 (11.1)  age matched | N/A | N/A | 1.51 (0.4)  1.60 (0.4)  1.55 (0.3)  ns | N/A | 0.322 (0.029)  0.322 (0.066)  0.311  (0.025)  ns | 0.473 (0.097)  0.479 (0.079)  0.599 (0.048)  * | |  | | N/A | +  weak group differences |
| Perry et al. [65] | Arthritis or chronic myofascial pain | Arthritis patient  myofascial pain group  normal control  Arthritis vs normal  Myofascial vs normal | 19 (5 males)  17 (0 males)  38 (22 males) | 46.5 (3.4)  43.8 (3.0)  34.1 (1.6) | 36.3 (2.4)  ${mm}^{2}$/s  36.1 (2.3)  ${mm}^{2}$/s  41.2 (1.3)  ${mm}^{2}$/s  pupil area per second  *  * | N/A | N/A | 37.8 (1.7)  38.6 (2.3)  41.2 (1.3)  *  * | N/A | N/A/s/s/s | | N/A | | -skin conductance  -HRV  (during mental arithmetic and Valsalva maneuver)  the result suggest decreased tonic peripheral parasympathetic tone in both groups and a decreased parasympathetic reaction in arthritis | + |
| Pozzessere et al. [71] | Multiple sclerosis (MS) | all MS patient  all control  MS patient baseline pupil ≤ 6mm  control  baseline pupil ≤ 6mm  MS patient 6mm<baseline pupil≤ 6.95mm  control 6mm<baseline pupil≤ 6.95mm  MS patient baseline pupil> 6.95mm  control  baseline pupil> 6.95mm  all MS vs all normal  MS vs normal (baseline pupil ≤6)  MS vs normal (6mm<baseline pupil≤6.95mm  MS vs normal (baseline pupil>6.95mm | 36 (15 males)  36 (15 males)  12 (5 males)  12  14 (6 males)  13  10 (4 males)  11 | 36.5 (6.37)  37.1 (7.20)  43.1 (3.90)  35.9 (7.00)  30.4 (5.4) | 2.84 (0.67)  3.40 (0.43)  2.52 (0.50)  3.20 (0.27)  2.93 (0.65)  3.59 (0.38)  3.12 (0.34)  3.41 (0.52)  *  *  *  ns | N/A | N/A | 23.2 (5.4)  29.1 (5.1)  23.1 (6.2)  30.5 (4.4)  23.9 (5.9)  30.7 (2.7)  23.2 (3.9)  27.6 (5.6)  *  *  *  ns | 0.276 (0.025)  0.269 (0.017)  0.2934 (0.0357)  0.2714 (0.0108)  0.2672 (0.0211)  0.2734 (0.0229)  0.2739 (0.0172)  0.2727 (0.0182)  ns  ns  ns  ns | N/A | | N/A | | N/A | + |
| Yuan et al. [44] | Diabetic autonomic neuropathy (DAN)  &  autonomic dysfunction from other causes (non-DAN) | DAN  non-DAN  DAN vs non-DAN | 40 (21 males)  40 (21 males) | 62.4 (28-89)  62.5 (26-89) | 2.8 (1.4)  3.6 (1.1)  *^ | N/A | 0.9 (0.5)  1.2 (0.4)  * | N/A | N/A | N/A | | N/A | | -QSART  -${HR}_{DB}$ range  -blood pressure  -HR during Valsalva maneuver  all of the measurements above contributed to the CASS score | + |
| Fotiou et al. [18] | Alzheimer’s Disease (AD) and Parkinson’s Disease (PD) (with and without cognitive disorder) | AD patient  PD patient  PDNoCog group  PDCog group  normal control  AD vs normal  PDNoCog vs normal  PDCog vs normal  PDNoCog vs PDCog | 23 (10 males)  22 (10 males)  11 (5 males)  11 (5 males)  23 (10 males) | 72.4 (5.4)  72.7 (7.3)  72.1 (7.1)  73.4 (7.6)  71.9 (8.5) | result were showed by AUC value as an index for classification accuracy | | | | | | | T2  time for MCV  *  ns  ns  ns | T3  time for ACA  * |  |  |
|  |  |  |  |  | *  *  *  * | *  *  *  * | *  ns  *  * | *  N/A  N/A  N/A | *  ns  *  ns | |  |  |  |  |  |
| Hakerem et al. [53] | Acute or chronic schizophrenia | acute  chronic  normal  normal vs acute  normal vs chronic  acute vs chronic | 37 (16 males)  41 (27 males)  22 (13 males) | 28  30  22 | ns  ns  ns | N/A | ns  ns  ns | N/A | ns  ns  ns | *  *  ns | | Flatn-ess of curve  ns  ns  ns | | N/A | + |
| Dutsch et al. [40] | Type 2 Diabetes mellitus (DM) | DM patient  DM patient with PN (peripheral neuropathy)  DM patient without PN  DM patient with CAN (cardiovascular neuropathy)  DM patient without CAN  normal  all DM vs normal  PN vs non PN  CAN vs non CAN | 36 (20 males)  20  16  20  16  36 (20 males) | 57.3 (23.5)  55.1 (19.7) | 3.90 (1.36)  3.76 (0.96)  3.79 (0.87)  3.67 (0.95)  3.88 (0.79)  4.49 (0.96)  *  ns  ns | N/A | 1.30 (0.36)  1.25 (0.36)  1.37 (0.37)  1.28 (0.35)  1.33 (0.34)  1.55 (0.31)  *  ns  ns | N/A | N/A | N/A | | N/A | | -HRV  -blood pressure  help to identify CAN  -nerve conduction  -vibratory thresholds  -thermal thresholds  help to identify PN | + |
| Kuroda et al. [38] | Borderline Diabetes Mellitus (B-DM) and early stage Diabetes Mellitus (E-DM) | B-DM group 1  B-DM group 2  B-DM group 3  E-DM group  control  B-DM goup1 vs normal  B-DM goup2 vs normal  B-DM goup3 vs normal  E-DM vs normal | 4 (4 males)  6 (6 males)  7 (7 males)  6 (6 males)  9 (9 males) | 48.5 (3.1)  48.7 (1.2)  48.9 (1.7)  46.7 (2.1)  50.0 (1.5) | unit for pupil size is ${mm}^{2}$ instead of mm | | | | | | | | | -HRV  no differences among groups | + |
|  |  |  |  |  | 32.3 (3.0)  29.2 (1.8)  38.7 (2.1)  ns  ns  ns  * | N/A | 10.9 (0.9)  15.1 (0.9)  ns  ns  ns  * | 40.4 (4.2)  39.8 (2.2)  45.7 (3.5)  59.1 (2.9)  54.0 (2.2)  *  *  *  ns | 0.2875 (0.0088)  0.293 (0.0043)  0.2948  (0.0071)  0.287 (0.0087)  0.3009 (0.0059)  ns  ns  ns  ns | N/A | | N/A | |  |  |
| Harle et al. [59] | Migraine | migraine patient  healthy control  migraine vs control  inter-eye difference migraine vs normal | 20 (3 males)  16 (2 males) | 37.3 range (32-42)  36.4  range (32-40) | N/A | N/A | ns  ns | N/A | 0.638  0.631  ns  * | N/A | | anisocoria at baseline and lateraliasation of headache are significantly correlated | | N/A | + |
| Kang et al. [29] | Parkinson’s disease (PD) | PD patient  healthy control  PD vs normal | 15 (11 males)  18 (9 males) | 65.7 (12.3)  60.3 (13.5) | N/A | N/A | N/A | N/A | N/A | N/A | | constriction velocity (velocity to minimum diameter)  ns | | -HRV  HF of HRV is significantly lower in PD patient comparing to control group | 0 |
| Dutsch et al. [74] | Familial dysautonomia | FD patient  healthy control  FD vs normal | 14 (8 males)  14 (6 males) | 18.7 (8.4)  20.4 (6.3) | 3.75 (1.09)  5.80 (0.59)  * | N/A | 1.34 (0.21)  1.86 (0.14)  * | 22.74 (7.11)  30.76 (3.50)  * | N/A | N/A | | N/A | | N/A | + |
| Fotiou et al. [31] | Alzheimer’s disease (AD) | AD medication free  AD under medication  healthy control  AD medication free vs normal  AD medication free vs AD medication  AD medication vs normal | 5  5  5 | The patient group and control group are age and gender matched | N/A | N/A | 0.51 (0.04)  0.39 (0.09)  0.36 (0.09)  ns  ns  ns | N/A | 0.20 (0.03)  0.30 (0.05)  0.18 (0.07)  ns  ns  ns | N/A | | latency to maximum constriction  *  ns  ns | | N/A | + |
| Inoue and Uemura [68] | Meniere’s disease (MD) | MD in Attack or quasiattack stage  MD in interval stage  normal control  MD in Attack or quasiattack stage vs normal  MD in interval stage vs normal | 10  22  20 (14 males) | N/A | values shown as differences between the left and right PLR | | | | | | | | | N/A | +  This study only examined the differences between the left and right PLR, which is different than other type of PLR analysis. While MCV was found to be the most sensitive indexes. |
|  |  |  |  |  | 0.78 (0.41)  0.39 (0.41)  0.23 (0.15)  *  ns | 0.22 (0.13)  0.16 (0.20)  0.11 (0.08)  ns  ns | N/A | N/A | 0.013 (0.015)  0.013 (0.012)  0.007 (0.009)  ns  ns | N/A | | N/A | |  |  |
| Pozzessere et al. [37] | Aging | Group 1 (age 15-29)  Group 2 (age 30-44)  Group 3 (age 45-59)  Group 4 (age 60-75)  Group 1 vs Group 4  Group 2 vs Group 4  Group 3 vs Group 4 | 18 (9 males)  12 (6 males)  12 (6 males)  10 (5 males) | 24.8 (3.3)  36.9 (5.5)  49.4 (4.4)  66.0 (5.4) | 3.554 (0.365)  3.416 (0.732)  3.412 (0.307)  2.852 (0.812)  *  ns  ns | N/A | 1.85 (0.27)  1.77 (0.35)  1.81 (0.27)  1.49 (0.25)  *  *  * | 29.604 (5.590)  28.177 (5.157)  30.906 (4.354)  23.765 (4.053)  *  *  * | 0.2733 (0.0179)  0.2763 (0.0226)  0.2683 (0.0164)  0.2855 (0.0169)  ns  ns  ns | 0.523 (0.070)  0.522 (0.052)  0.536 (0.056)  0.557 (0.149)  ns  ns  ns | | N/A | | N/A | + |
| Micieli et al. [34] | Multiple system atrophy (MSA): striatonigral degeneration (SND)  or  olivopontoce-rebellar (OPCA) | MSA  Parkinson’s Disease  normal control  MSA vs normal  MSA vs PD  PD vs normal | 14 (11 males)  23 (15 males)  26 (20 males) | 59.1 (7.6)  58.5 (9.9)  54.2 (11.3) | N/A | N/A | *  *  * | N/A | *  *  * | *  *  * | | N/A | | N/A | + |
| Ferrari et al. [41] | Diabetes Mellitus (DM) with or without cardiovascular autonomic neuropathy (CAN) | DM patient without CAN  DM patient with CAN  normal control  DM without CAN vs normal  DM with CAN vs normal  DM with CAN vs DM without CAN | 16 (9 type 1, 7 type 2; 15 males)  8 (3 type 1, 5 type 2; 7 males)  16 (9 males) | 50 (11)  51 (5)  44 (13) | unit in pixel/s  10.28 (3.49)  7.87 (3.48)  14.57 (5.32)  *  *  ns | N/A | unit in pixel  7.40 (1.90)  5.35 (2.14)  9.62 (1.99)  *  *  ns | N/A | 0.26 (0.070)  0.29 (0.100)  0.20 (0.078)  ns  *  ns | ns  ns  ns | | N/A | | -HRV  -blood pressure  to classify patient group with CAN or without CAN  Systolic blood pressure result showed significant differences between normal control and patients. Diastolic blood pressure was larger in diabetes with CAN in comparison with controls | + |
| Bitsios et al. [22] | Aging | young group  old group  young vs old | 12 (8 males)  14 (6 males) | 19.5 range (19-26)  69 range (61-79) | * | N/A | * | N/A | *only in high intensity | N/A | | N/A | | N/A | + |
| Bittner et al. [32] | AD | AD patients  mild cognitive impairment (MCI)  normal control  AD vs normal  MCI vs normal  AD vs MCI | 66  42  44 | 74.4 (7.5)  69.8 (8.4)  66.4 (8.3) | N/A | N/A | 0.93 (0.31)  0.94 (0.32)  1.04 (0.28)  ns  ns  ns | 0.197 (0.070)  0.178 (0.069)  0.186 (0.060)  ns  ns  ns | 0.282 (0.031)  0.285 (0.029)  0.275 (0.022)  ns  ns  ns | N/A | | N/A | | N/A | 0 |
| Pfeifer et al. [42] | Diabetes Mellitus (DM) | DM patients  normal control  DM vs normal | 25  24 | 47 (3) range: 21-64  42 (4) range: 22-79 | 0.30 (0.02)  0.35 (0.02)  ns |  | 2.19 (0.13)  2.67 (0.14)  * | N/A | 0.418 (0.005)  0.355 (0.004)  * | N/A | | N/A | | N/A | + |
| Levy et al. [43] | Diabetes Mellitus (DM) | DM patient  (half type1 and half type 2)  normal control  DM vs normal | 85 (53 males)  67 (32 males) | 44 (15)  range: 18-66  42 (12)  range: 19-67 | 3.82 (1.12)  5.68 (1.55)  ns | N/A | 1.59 (0.51)  1.49 (0.47)  ns | 32.91 (9.92)  33.02 (8.41)  ns | 0.25 (0.06)  0.23 (0.05)  * | 0.79  range (0.67-0.79)  0.78  range (0.62-1.04)  ns | | N/A | | -Marstock thermal thresholds  -Vibration perception thresholds  -HRV  -blood pressure  -sweat tests  ACA and MCV correlated with R-R interval, postural systolic and diastolic changes in blood pressure | +  only latency was significantly different between DM patients and normal controls |
| Barendregt et al. [66] | rheumatoid arthritis (RH) with ocular dryness | Group 1 (RH patient with ocular dryness)  Group 2 (RH patient without ocular dryness)  Group 3 (normal control)  Group 1 vs Group 2  Group 1 vs Group 3 | 18 (0 male)  18 (0 male)  33 (0 male) | 62  range: 32-82  55  range: 24-75  57  range: 26-82 | N/A | N/A | N/A | N/A | 0.260  0.245  0.250  *  * | 0.350  0.320  0.325  *  * | | N/A | | N/A | + |
| Pfeifer et al. [46] | non-insulin-dependent diabetes (NIDD) | untreated NIDD patient  normal control  NIDD vs normal | 38 (38 males)  58 (58 males) | 57 (1)  range: 30-68  42 (2)  range: 21-78 | N/A | N/A | N/A | N/A | * | N/A | | N/A | | -R-R variation as an index of cardiac parasympathetic activity  significantly correlated with Latency in NIDD patient group | + |
| Piha et al. [49] | obesity | (identical twins)  obese  non-obese  obese vs non-obese | 15  15 | N/A | 7.35 (2.50)  7.57 (2.00)  ns | N/A | 2.05 (0.57)  2.02 (0.38)  ns | 41.1 (7.2)  40.1 (4.2)  ns | N/A | 0.82 (0.08)  0.80 (0.09)  ns | | N/A | | -blood pressure responses during the Valsalva manoeuvre  -HRV during deep breathing test  -active orthostatic test  -isometric handgrip test | 0 |
| Baum et al. [50] | childhood obesity | obese  normal weight  obese vs normal weight | 90 (45 males)  59 (34 males) | male: 12.2 (2.3) range: 7.9-17.9  female:  11.9 (2.7)  range: 7.4-17.5)  male: 12.4 (2.6)  range: 8.0-17.3)  female: 11.5 (2.6)  range:  7.4-17.5 | N/A | N/A | ns | N/A | ns | N/A | | constriction velocity  ns | |  | 0 |
| Bremner and Smith [75] | generalized autonomic neuropathy which sub-divided into specific diseases | amyloidosis  multiple system atrophy (MSA)  pure autonomic failure (PAF)  diabetes  normal control  MSA vs normal  PAF vs normal  normal vs amyloidosis  normal vs diabetes | 21 (14 males)  38 (26 males)  33 (15 males)  29 (16 males)  315 (172 males) | median age: 57  range:  31-70  median age: 58  range:  40-79  median age: 60  range: 30-80  median age: 46  range: 19-76  median age: 42 (17-82) | result was based on the comparison of percentage of abnormality within different groups % | | | | | | | | | N/A | + |
|  |  |  |  |  | N/A | N/A | 7.9  9.7  28.6  33.3  2.0  ns  ns  *  * | N/A | N/A | N/A | | N/A | |  |  |

Note:

The assumption is:

Pupil light reflex is an effective method to measure parasympathetic activity. If a disease is related to parasympathetic dysfunction, then the patients with this type of disease should reflect a different (attenuated) PLR than the comparison group.

N/A: not applicable

ns: not significant

* : significant

^*: most sensitive parameters as claimed by the author(s) of that study

+: positive relationship to the assumption

-: negative relationship to the assumption

0: no difference between groups

Only the parameters considered to be related to parasympathetic nervous system function were included (PNS only)

For PLR parameters:

MCV: maximum constriction velocity

MCA: maximum constriction acceleration

ACA: absolute constriction amplitude

RCA: relative constriction amplitude
